# Comparative Evaluation of the Remineralizing Effects and Surface Micro hardness of Glass Ionomer Cements Containing Bioactive Glass (S53P4):An *in vitro* Study

**DOI:** 10.5005/jp-journals-10005-1057

**Published:** 2010-08-17

**Authors:** AR Prabhakar, Jibi Paul M, N Basappa

**Affiliations:** 1 Professor and Head, Department of Pedodontics and Preventive Dentistry, Bapuji Dental College and Hospital, Davangere Karnataka, India; 2 Postgraduate Student, Department of Pedodontics and Preventive Dentistry, Bapuji Dental College and Hospital, Davangere Karnataka, India; 3 Professor, Department of Pedodontics and Preventive Dentistry, Bapuji Dental College and Hospital, Davangere Karnataka, India

**Keywords:** Demineralization, glass ionomer cements, bioactive glass, remineralization, surface microhardness.

## Abstract

Dental cements including the glass ionomer cement (GIC) have found widespread use in restoring tooth structures. In this study, modifications of glass ionomer cements (GICs) were made by adding bioactive glass (BAG) to GIC to obtain bioactive restorative materials. This study used polarized light microscopy (PLM) to examine the remineralization effects of the study materials on dentin. It also evaluated the Vickers microhardness of the experimental materials. Experimental glass ionomer cement (GIC)-BAG materials were made by mixing 10 wt% of BAG particles with conventional cure and resin-modified GIC powders. Class V restorations were made in 80 extracted mandibular teeth which included 4 groups of 20 teeth each. 100 |jm sections of the teeth were examined under polarized light microscope after undergoing pH cycling. Materials were also processed into 80 cylindrical specimens and immersed in water for 7 and 30 days before mechanical tests. Resin-modified GIC containing BAG showed a thick uniform layer of mineralization on the restoration-dentin interface. The conventional cure GIC-based materials had higher surface microhardness than the resin-modified materials.

*Significance:* The addition of BAG to GIC compromises the mechanical properties of the materials to some extent. Thus, their clinical use ought to be restricted to applications where their bioactivity can be beneficial, such as root surface fillings and liners in dentistry.

## INTRODUCTION

Glass ionomer cements (GICs) are widely used in restorative dentistry.^1^ In dentistry, one of their advantages over other restorative materials is that they can be placed into tooth cavities without an additional bonding agent.^2^ They also possess a fluoride-releasing property^3^ and are relatively biocompatible with the pulp.^1^ Adhesive property and fluoride release of glass ionomer cement contribute to reduce the rate of secondary caries both in filled teeth and on the enamel surfaces of adj acent teeth.^4,5^ Although, widely used as dental cements, GICs have some disadvantages. One problem is that they do not always bond sufficiently to enamel and dentin. The mechanical strength decreased significantly when the paste is exposed to saliva at the initial stage of setting. To overcome this drawback, conventional glass ionomer cement was modified by water soluble resin.^6^ These resin modified glass ionomer cements (RMGICs) provided improved mechanical properties and adhesion, better esthetics, easier application with reduced moisture sensitivity and immediate light cure after placement.^7^ They were mostly found to have a potential for releasing fluoride in equivalent amounts as conventional glass ionomer cements.^8^

Bioactive glasses (BAG) are surface-active glasses with which bone minerals are able to bond chemically.^9^ The components in bioactive glass are basically oxides of calcium, sodium, phosphorus, and silicon at certain weight ratios^10-13^ that provide the material with surface activity.^14^ The bioactive nature of BAG is related to their ability to form a bone-like apatite layer on their surfaces in the body environment.^9^ In addition to being biocompatible, bioactive glasses bond to and stimulate the regeneration of bone.^15,16^ So far, most studies of bioactive glass have been focused on orthopedic bone research.^10-13^ However, there is growing interest in the application of bioactive glass in dentistry, especially for dentin mineralization or remineralization.^17,18^ Several components involved in the body environment and the oral environment are similar for the interaction between bioactive glasses and dentin. Saliva has a comparable ionic composition to plasma, and bone and dentin are analogous in composition, if not in microstructure. Additionally, bone and dentin have similarities in their processes for formation; a main difference is that dentin is not vascularized.^16^ Bioactive glass is considered a break through advance in remineralization technology. This is because the current standard treatment for tooth remineralization and prevention of decay is a slow acting and is dependent on adequate saliva as a source of calcium and phosphorus.^19,20^

Although, glass ionomer cement contains both calcium and phosphate, it does not show any bioactivity. It would be definitely an advantage of glass ionomer cement if it could possess bioactivity^21^ because currently, there is a trend for the development of biomaterials that have therapeutic or bioactive functions, in addition to their inherent properties.^22^ Matsuya et al^23^ reported a new glass ionomer cement based on the bioactive CaO - P_2_O_5_ - SiO_2_ (- MgO) glass and polyacrylic acid. They investigated its setting process using Fourie-transform infrared (FT-IR) and magic angle spinning-nuclear magnetic resonance (MAS-NMR) spectroscopies. They found that Ca^2+^ was released from the bioactive glass to form carboxylate salt and the degree of polymerization in the silicate network increased. The setting mechanism of the new cement was essentially the same as that of the conventional glass ionomer cement, which is, an acid-base reaction between the basic glass and the polymeric acid.

When selecting a restorative material, one of the main considerations is its mechanical properties. As a restorative material is used to replace missing tooth structure, it needs to be strong enough to withstand the forces associated with mastication. Hardness test can be used to evaluate these mechanical properties. Although, there are studies available comparing microhardness between GIC and RMGIC, little, however, has been conducted on the alteration of the surface micro microhardness of these materials containing BAG which have been placed in a liquid medium. Thus the aim of the present study was to evaluate and compare the remineral-izing effects of glass ionomer cement-containing bioactive glass in comparison with conventional and resin-modified glass ionomer cements. A further aim was to explore the surface microhardness of the material surfaces.

## MATERIALS AND METHODS

### Evaluation of Remineralization Effects

In vitro Caries Production

**Fig. 1:**
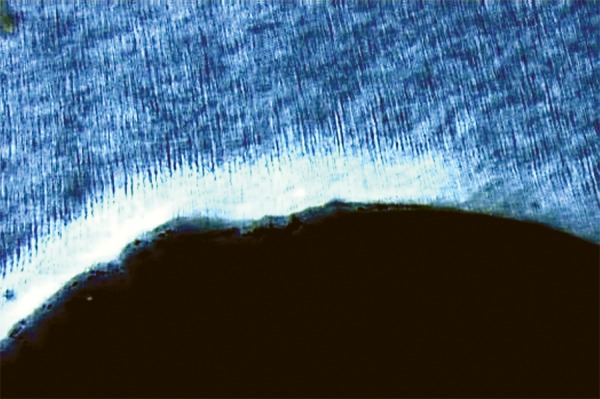
Demineralized area

**Fig. 2:**
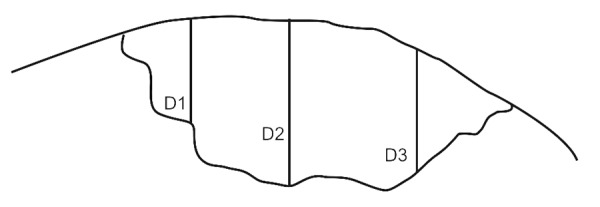
Measurement of carious lesion at 3 sites

Eighty permanent mandibular premolars extracted for orthodontic reasons, were used throughout this study.^24^ Only teeth that were free of caries and restorations and showed no evidence of white spots or cracks on buccal or lingual surfaces were selected.^25^ After extraction, the teeth were polished with pumice on a prophylactic brush; steam autoclaved and immediately stored in cold distilled water at 4°C for 1 to 2 months before testing. Standardized class V cavities, one on the buccal and one on the lingual surface of each tooth were prepared with a high speed diamond flat end cylinder bur (No: 108008, 0.8 mm, Horico^®^, Germany) using water as a coolant. The cavity preparation was 3 mm wide, 2 mm high and 1.5 mm deep^25^ and it was placed parallel to the cementoenamel junction (CEJ) with the preparation extending 1 mm above the CEJ.^26^ The bur was replaced after every fifth preparation. Each cavity was measured with a William’s graduated probe to ensure uniform size.^27^ All cavities were cleaned with an air/water mixture from a triple syringe and dried with air. The teeth were then randomly divided into 4 experimental groups of 20 teeth each. They were covered with 2 coats of acid-resistant nail varnish except for window which included the cavity and a 2 mm rim of sound tooth structure surrounding the restorations. The first coat was left to dry at room temperature for 3 to 4 hours before a second coat was applied.^28^ Artificial caries like lesions were created on the exposed cavities by suspending all four groups of teeth in an artificial caries system for 2 days (50 ml per sample). The caries solution consisted of 2.2 mM Ca^+2^, 2.2 mM PO_4_^-3^, 50 mM acetic acid at a pH of 4.4. The solution was kept at a temperature of 37°C, under constant circulation.^27^ After 2 days, teeth were removed from the artificial caries system. Each tooth was sectioned longitudinally in the occlusogingival direction to get one buccal and one lingual half in which one half was used as control and the other as a test specimen randomly, where the test specimens were preserved to be used later. Control specimens were mounted on acrylic blocks for sectioning. A section of 100 |j,m thickness was obtained by cutting through the center of the cavity using a hard tissue microtome (Leica SP 1600, Leica Microsystems, Nussloch, Germany). Sections were washed with deionized water and oriented longitudinally on glass cover slides. The sections were imbibed with DPX mounting medium for evaluation under polarized light microscopy using an Olympus dual stage polarized light microscope (model BX-51, Dualmont Corporation, Minneapolis, Minn). Sections were photographed under maximum illumination. Photomicrographs were made at X10 magnification. The demineralized areas (Fig. 1) were quantified with a computerized imaging system Image Pro-Plus^27^ (Measure and Clarity, Media Cybernetics, Inc., Bethesda, MO, USA). The artificial lesion was quantified at 3 points. Lesion depth was measured from the surface of the lesion to the depth of the lesion, at D1, D2 and D3 (Fig. 2).^29^ Lesion depth for each section (in μ) was taken as the average of the three representative measurements from the surface of the lesion to the depth of the lesion.

Preparation of Samples for Remineralization

Two different commercially available GICs were used: Conventional cure GIC (GC Fuji II, GC Corporation, Tokyo, Japan) and Resin-modified light cure GIC (Fuji II LC, GC Corporation, Tokyo, Japan). These formed the first and second experimental groups (GI and LCGI). These materials consisted of powder and liquid. A commercially available BAG (S53P4 bioactive glass Frit. Size: < 53 μm, MO - SCI^®^ health care, Rolla, MO, USA) was used. The composition of the BAG by weight was SiO_2_ 53%, Na_2_O 23%, CaO 20% and P_2_O_5_ 4%, while the particle size was < 53 μm. The last two experimental material powders were made by mixing 10 wt% BAG particles with GIC and RMGIC powders (GI10BAG and LC10BAG). A description of the total GIC/BAG powder ratio and the powder to liquid ratio is given in Table 1.

**Fig. 3:**
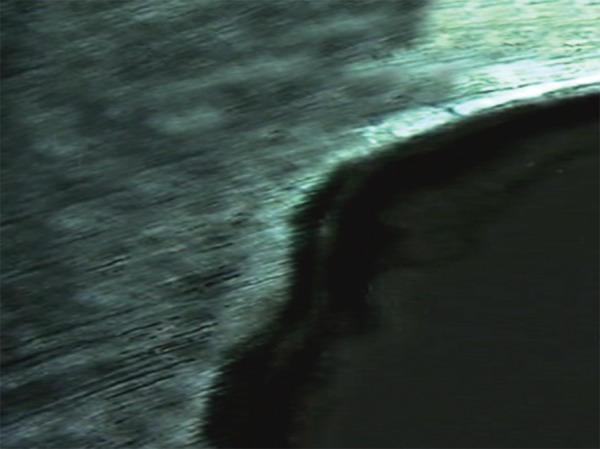
Remineralization in Group I

**Fig. 4:**
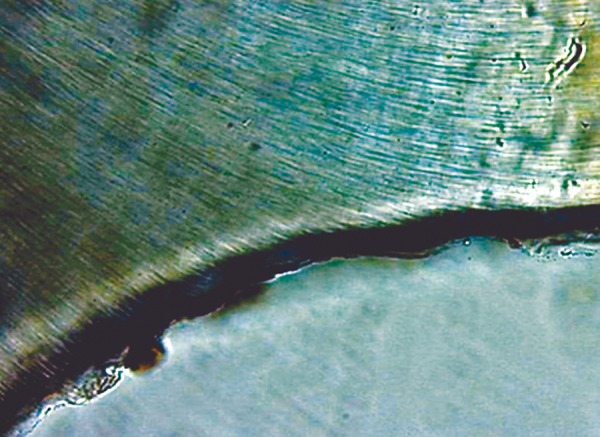
Remineralization in Group II

**Fig. 5:**
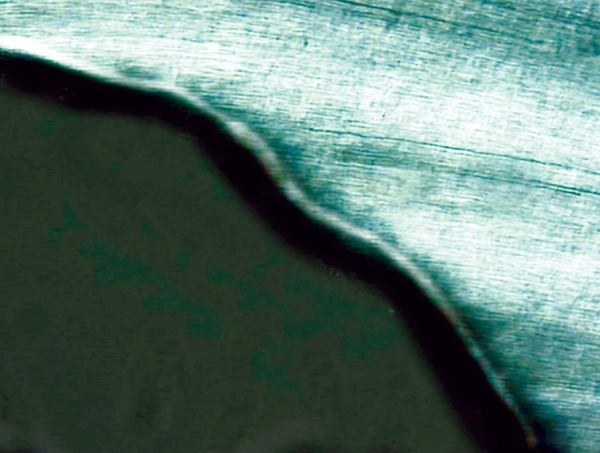
Remineralization in Group III

**Fig. 6:**
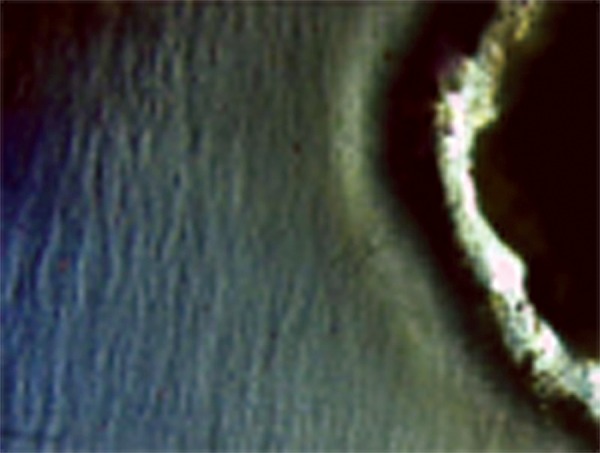
Remineralization in Group IV

The cement powders were mixed with poly acrylic-acid of GIC and a dimethacrylate resin-poly acrylic-acid mixture of RMGIC according to the manufacturers’ instructions. The cavities of teeth segments were restored with respective experimental materials. The specimens of LCGI and LC10BAG were polymerized with a visible light curing device (Bee Cool. Plus TOP light LED curing) (470 nm wavelength, light intensity 690 mW/cm^2^) for 20 s. All the specimens were prepared at room temperature (21 ± 1°C), in 55% relative humidity.^30^ The restored teeth segments were stored in humid environment at 37 ± 1°C for 24 hours. After that, the excess restorative material was removed and polished.^31^ These restored tooth specimens were subjected to a daily cyclic treatment regime which involved exposing the specimen to remineralizing-demineralizing solutions. The remineralizing solution used contained 2 mM calcium chloride and 2 mM sodium dihydrogen or-thophosphate. The pH was adjusted to 6.8 by the addition of 0.1 M sodium hydroxide. The demineralizing solution was the same as that used for lesion creation. Each specimen was immersed in 10 ml of remineralizing solution for 20 hours at 37°C, removed and washed with deionized water and then immersed in 10 ml of demineralizing solution within another vial for 4 hours at 37°C. The cycling program was carried out for 28 days.^32^ At the end of the 28th cycling period, the specimens were removed from the pH cycling regime and were mounted on acrylic blocks. The 100 u.m sections from individual specimens were oriented longitudinally on the glass cover slides for evaluation under polarized microscope. The remineralized lesions (Figs 3 to 6) were again quantified using imaging system, Image Pro-Plus, as described for demineralization.

**Table 1:** **Table 1:** Weight ratio (%) of conventional GIC (GI), resin modified GIC (LCGI) and bioactive glass (BAG) particles, and powder-to-liquid ratios (P/L) in the experimental materials when using the level scoops recommended by the manufacturer of the GICs used in this experiment

*Group* *(g/g)*		*GI*		*LCGI*		*BAG*		*P/L*	
GI		100						2.7	
GI10BAG		90				10		1.7	
LCGI				100				3.2	
LC10BAG				90		10		2.2	

### Evaluation of Surface Microhardness

A total of 80 cylindrical specimens of 20 each in a group were made from the same 4 experimental materials. The test specimens were made by placing the mixed materials into standardized cylindrical brass molds (diameter, 10 mm; height, 1.5 mm),^33^ then slightly overfilling them and gently compressing them between two glass plates. The specimens made from LCGI and LC10BAG were polymerized with a visible light curing device (Bee Cool. Plus TOP light LED curing) (470 nm wavelength, light intensity 690 mW/cm^2^) for 20 s on both ends of the specimen. All the specimens were prepared at room temperature (21 ± 1°C), in 55% relative humidity. The specimens were gently removed from the molds, stored at 37°C for 1 hour, and immersed individually in test tubes in 20 ml of deionized water in an incubator at 37°C. Immersion times were 7 days and 30 days. After 7 days of immersion, 10 specimens from each group were randomly selected and subjected to microhard-ness measurements. The same procedure was repeated on the remaining specimens at the end of 30 days.

**Fig. 7:**
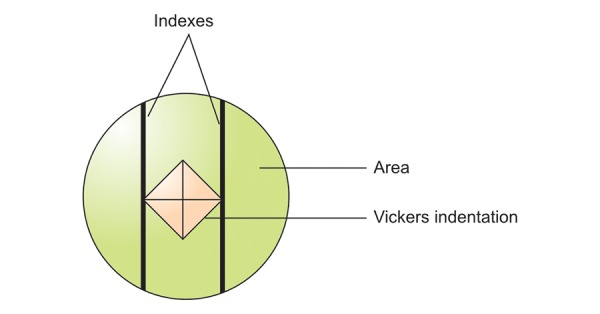
Vickers hardness measurement

The microhardness measurements were carried out using a microhardness tester (HMV-2000 SHIMADZU, Tokyo, Japan). The indentations were made within 5 s from the loading (0.25 N) for all specimens, to eliminate possible plastic deformation of the polymer matrix of resin-modified GICs that would influence the surface microhardness values. For each test specimen, the values were read referring to the size of the greater diagonal (Fig. 7). The values were transformed into Vickers hardness number.^34^ Surface microhardness was calculated using the following formula: VH = 1.854 × F × 10^3^/d^2^ where, F is the applied test load (N) and d is the average of the indentation diagonals (mm).^35^

The results from observations of both experiments were tabulated and statistically analyzed. Results were expressed as Mean ± Standard deviation, range and percentage changes. Paired t-test was performed to analyze the changes in the depth of demineralization and remineralization. One way ANOVA was used for multiple group comparison followed by Post-Hoc Tukey’s test for pairwise comparisons. For all the tests, a p-value of 0.05 or less was considered for statistical significance.

## RESULTS

### Remineralization

**Fig. 8:**
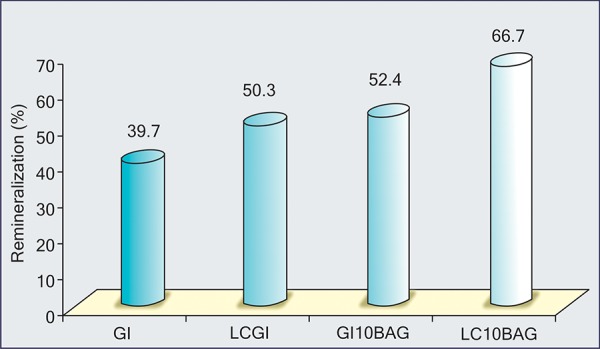
The comparison of the percentage of remineralization among the various experimental groups

Results observed a definite amount of remineraliza-tion with all the experimental groups. GI10BAG and LC10BAG showed higher remineralization effects than the resin modified LCGI and conventional GI, among which again highest remineralization was observed with LC10BAG (p < 0.01) (Fig. 8). Table 2 summarizes the difference in de- and remineralization for the various control and experimental groups.

**Table 2:** **Table 2:** Descriptive analysis showing the mean and standard deviation and the significant (p) value of difference in the demineraliza-tion and remineralization among the various control and experimental groups

*Groups*		*Number of* * samples*		*Demineralization* * (Control group)*		*Remineralization* * (Experimental group)*		*Mean* *difference*		*Significance*			
										*t-value*		*p-value*	
**Group I** GI		20		33.1 ± 13.0		13.0 ± 5.8		20.1		10.5		< 0.001	
**Group II** LCGI		20		44.9 ± 15.9		23.2 ± 12.2		21.7		11.7		< 0.001	
**Group III** GI10BAG		20		24.4 ± 12.8		17.7 ± 6.2		6.7		8.9		< 0.001	
**Group IV** LC10BAG		20		35.9 ± 12.7		24.7 ± 11.6		11.2		12.8		< 0.001	

### Surface Microhardness

**Fig. 9:**
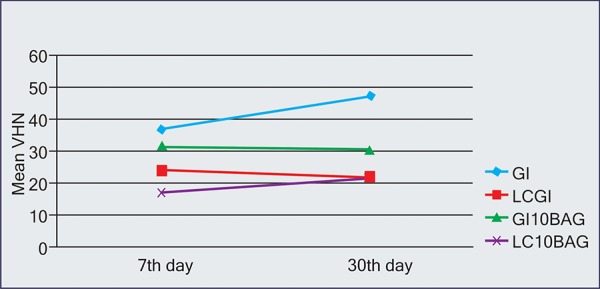
The comparison of surface microhardness among various experimental groups at 7th day and 30th day

Table 3 summarizes the surface microhardness values of various experimental groups at 7th day and 30th day. As shown in the table, conventional cure materials GI and GI10BAG showed generally higher VH values than the resin modified light-curing LCGI and LC10BAG. The VH of GI increased during immersion, and was clearly higher than that of the materials GI10BAG, LCGI and LC10BAG (p < 0.01). Also for LC10BAG, the VH increased during immersion; while the materials GI10BAG and LCGI showed decreasing VH values (Fig. 9).

**Table 3:** **Table 3:** Descriptive statistics showing the intragroup comparison of the mean and standard deviation and the significance p-value of difference in surface microhardness among the various experimental groups at 7th day and 30th day

*Groups*		*7th day*		*30th day*		*Mean difference*		*7th day vs 30th day*			
								*t-value*		*p-value*	
**Group I** GI		36.8 ± 4.6		47.7 ± 5.4		10.9 ↑		4.83		< 0.01,S	
**Group II** LCGI		24.3 ± 2.8		22.0 ± 2.4		2.3 ↓		2.01		0.06, NS	
**Group III** GI10BAG		32.0 ± 3.5		30.6 ± 3.2		1.4 ↓		0.92		0.37, NS	
**Group IV** LC10BAG		17.2 ± 2.7		21.3 ± 2.1		4.1 ↑		3.74		< 0.01,S	

## DISCUSSION

Dentin accounts for the greatest part of the dental hard substance. Odontoblasts orchestrate mineralization processes in dentin, not only during dentinogenesis, but also after teeth have been formed.^36^ The current consensus is that caries beyond the dentinoenamel junction (DEJ) should be treated with restorations, and lesions up to that point should receive extrapreventive care. However, it was never studied whether deep lesions, extending into dentine, can be remineralized if such lesions are subjected to a continuous remineraliza-tion scheme.^37^ Previous studies have shown that fluoride-releasing conventional GICs have high cariostatic effect.^33^ This is partly attributed to the enhancing effect of fluoride on calcium phosphate precipitation, hence remineralization,^38^ However, for net remineralization to occur adequate levels of calcium and phosphate ions must be available and this process is normally calcium phosphate limited.^39^

Recently, bioactive glass materials have been introduced in many fields of dentistry which is known to cause calcium phosphate precipitations in their environment. Previous studies^9^ have suggested that BAG could be used for rem-ineralizing damaged dentin. As BAG particles alone are easily displaced in a clinical environment, a suitable carrier or matrix material such as GIC is needed to facilitate its use in clinical settings. In this study, BAG was incorporated in a definite proportion into conventional and resin-modified GICs, and the materials were studied under *in vitro* conditions.

The first objective of this study was to test if glass ionomer cement containing bioactive glass produced rem-ineralization. In order to evaluate this, artificial caries like lesions were created in the cavities made on premolar teeth by subjecting it to chemical caries model. This was chosen because these *in vitro* chemical models provide information about the effects of caries preventive agents on the de- and remineralization dynamics at the surface and in the subsurface of the teeth.^40^ Ten Cate^41^ observed that partially demineralized crystallites must be present to act as a clean surface for mineral deposition for remineralization to occur. Thus, the demineralization phase is an important component of the remineralization process and in moderation promotes subsurface remineralization. In the absence of demineralization, the evaluation of a product’s remineralizing performance may not be relevant. After restoration of the cavities with experimental materials, again the teeth were subjected to a pH cycling model which involved exposing the specimen to alternating remineralizing-demineralizing solutions. It reproduces a dynamic situation, because dental caries represents a process of alternating demineralization and remineralization phenomena that are direct function of conditions that maintain a critical pH in the mouth.^42,43^ The incorporation of an intermittent acid attack into such protocols is worthy, because it has been suggested that the incorporation of further acid attack into an experimental protocol would improve the sensitivity of *in vitro* deminer-alization and remineralization studies.^44^

On quantifying the depth of remineralization in the experimental groups it was evident that they showed significant remineralization. In our study, the greatest degree of depth of remineralization was found in the LC10BAG group. Similar observations were made by Helena Yli-Urp et al,^17^ who examined the release of Si, Ca, P, and F from conventional GIC and resin-modified GIC containing different quantities of BAG also *in vitro* biomineralization of dentine. He observed that the release of Si increased with the increasing immersion time from the specimens containing BAG, whereas the amount of Ca and P decreased indicating *in vitro* bioactivity of the materials. RMGIC with BAG showed highest bioactivity. It also showed calcium phosphate (CaP) like precipitation on both the surface of the test specimens and on the dentin disks immersed with the material. Another similar study^19^ evaluating the remineralization potential of bioactive glass on artificially carious enamel and dentin using Raman spectroscopy indicated that bioactive glass has the potential for remineralizing artificially carious enamel and dentin. In our study, mineral depositions close to the restoration-dentin interface and in the deeper parts of dentin tubules, especially in the resin-modified GIC containing BAG, indicate that the materials can induce dentin mineralization *in vitro.* BAGs are considered efficient silica and calcium sources for biomineralization processes. According to Damen et al,^45,46^ silica on dentin acts as a heterogenic nucleation center for CaP precipitation. In addition, BAG releases high concentrations of Ca which increase the concentration of Ca ions in the vicinity of the material and enhance mineralization. When adsorbed silica is condensed on the dentin, there are still free silanol groups (SiOH) that are thought to act as CaP nucleation centers. It is also suggested that Ca released from the BAG increases the ionic activity product of apatite and thus promotes nucleation of CaP.^18,40^

Differences between the conventional cure and resin-modified GICs may be due to the hydrophilic nature of the polymer matrix of the resin modified GIC. Yiu and co-workers^47^ suggested that in the ion-rich polyalkeno-ate matrix, an osmotic gradient might exist that causes permeation of water across the GIC-dentin interface. This osmotic pressure probably led to the absorption of water in the polymer matrix of GICs, creating the aqueous conditions needed for BAG particles to react with the surrounding tissues. This reactivity is directly related to the amount of BAG used in the experimental materials. With conventional cure GICs, there is less water absorption, BAG is less reactive towards polyacrylic acid, and there is therefore less surface reaction on BAG particles.^9^ This is probably the biggest reason why resin-modified materials were more reactive in this study and why the results are well in line with other *in vitro* investigations.

GICs and RMGICs in our study also showed a significant amount of remineralization as supported by previous stud-ies.^48^ It has been reported that fluoride levels in the plaque are elevated substantially for months after a GIC filling has been placed^49^ but also silica released from the GIC restoration could have a mineralization promoting effect, as has been reported from crystal growth studies of hydroxyapatite.^45^ The zone of hyper mineralization observed in the tissue in contact with the restoration agrees with that in various other studies.^50^ From the observations made by Mitra,^51^ it has been proved that under clinical condition, fluoride released from RMGI would inhibit demineralization and enhance remineralization.

With regard to the second objective of this study, the surface hardness of the conventional cure GICs were found to be higher than those of the resin-modified materials. This result is in accordance with an earlier study.^52^ The effect of adding BAG particles to the results of the current study was interesting. In the conventional cure materials, the surface of GI was significantly harder than the surface of GI10BAG, while the hardness of GI10BAG decreased even though it was not statistically significant. Totally opposite results were found with the resin-modified GICs. During immersion, the surface of LC10BAG became harder than the surface of LCGI. It has been previously shown that aging time has no significant effect on the surface hardness of conventional GICs.^53^ The changes in surface hardness that were found in this study are most probably related to the reactivity of the BAG. In resin-modified GICs, dissolving ions precipitate mostly on the material surface, while in conventional cure materials, precipitation may also occur within the material itself.

The water absorption could also contribute to surface hardness values. The decrease in microhardness of GI10BAG suggests that the BAG particles might be only loosely attached to the GIC matrix. Thus, BAG particles probably acted as fillers that had not been adhered into the matrix of GIC. In an aqueous environment BAG begins to release ions, which precipitate on the glass surface. It is known that the polymer matrix of RMGICs is hydrophilic in nature and therefore, absorbs water over time. The absorbed water in the polymer matrix of GICs can allow surface reactions of BAG particles to occur. Earlier studies have shown that there are different thicknesses of reactive layer of glass particles in resin-modified GICs. Furthermore, the reactive layer gets thicker when the immersion time increases, thus leading to increase in microhardness. In a previous study by Yli-Urpo,^35^ it was found that the surface hardness of BAG containing RMGICs increased during water immersion.

In our study, adding BAG to GIC impaired its surface hardness to a minimal extent. As mentioned earlier, surface hardness correlates well to compressive strength and abrasion resistance. It should be noted that the results of the present study are valid only for the aqueous conditions. Dependent upon their environment, GICs can reveal very different behavior. If GICs are exposed to an acidic environment, degradation of GICs can be very crucial. Resin-modified GICs are in conditions more stable than conventional ones. However, in pure water, the stability of GICs is good.^54^

## CONCLUSIONS

The following conclusions were drawn from the study:

- Bioactive glass can enhance mineral formation in the dentin and it has potential as a filler component in mineralizing restorative materials.
- Incorporation of bioactive glass into glass ionomer cements enhanced their remineralization property.
- Resin-modified GIC containing BAG has significant potential in clinical applications where enhanced mineralization is expected.
- Incorporation of BAG into RMGIC improved its mechanical property in aqueous environment, although it was less than RMGIC.

Within the limitations of this study, it can be concluded that resin-modified GICs containing BAG are promising restorative materials for clinical use where an increase in dentin mineralization can be beneficial. The addition of BAG to GIC compromises the mechanical properties of the materials to some extent. Thus, their clinical use ought to be restricted to applications where their bioactivity can be beneficial, such as root surface fillings, base and liner materials in deep cavities, and in the treatment of hypersensitive dentin.
